# Clinical Evaluation of BD Veritor SARS-CoV-2 Point-of-Care Test Performance Compared to PCR-Based Testing and versus the Sofia 2 SARS Antigen Point-of-Care Test

**DOI:** 10.1128/JCM.02338-20

**Published:** 2020-12-17

**Authors:** Stephen Young, Stephanie N. Taylor, Catherine L. Cammarata, Katey G. Varnado, Celine Roger-Dalbert, Amanda Montano, Christen Griego-Fullbright, Cameron Burgard, Catherine Fernandez, Karen Eckert, Jeffrey C. Andrews, Huimiao Ren, Joseph Allen, Ronald Ackerman, Charles K. Cooper

**Affiliations:** a Tricore Reference Laboratory, Albuquerque, New Mexico, USA; b Louisiana State University Health Sciences Center, New Orleans, Louisiana, USA; c Becton, Dickinson and Company, BD Life Sciences—Integrated Diagnostic Solutions, Sparks, Maryland, USA; d Becton, Dickinson and Company, BD Life Sciences—Integrated Diagnostic Solutions, San Diego, California, USA; e STAT Research, Vandalia, Ohio, USA; f Comprehensive Clinical Research, LLC, West Palm Beach, Florida, USA; UNC School of Medicine

**Keywords:** COVID-19, SARS-CoV-2, Veritor test, point-of-care test, Sofia 2 test

## Abstract

The clinical performance of the BD Veritor System for Rapid Detection of SARS-CoV-2 nucleocapsid antigen (Veritor), a chromatographic immunoassay used for SARS-CoV-2 point-of-care testing, was evaluated using nasal specimens from individuals with COVID-19 symptoms. Two studies were completed to determine clinical performance. In the first study, nasal specimens and either nasopharyngeal or oropharyngeal specimens from 251 participants with COVID-19 symptoms (≤7 days from symptom onset [DSO], ≥18 years of age) were utilized to compare Veritor with the Lyra SARS-CoV-2 PCR assay (Lyra). In the second study, nasal specimens from 361 participants with COVID-19 symptoms (≤5 DSO, ≥18 years of age) were utilized to compare performance of Veritor to that of the Sofia 2 SARS Antigen FIA test (Sofia 2). The positive, negative, and overall percent agreement (PPA, NPA, and OPA, respectively) were the primary outcomes. In study 1, the PPA for Veritor, compared to Lyra, ranged from 81.8 to 87.5% across the 0 to 1 and 0 to 6 DSO ranges. In study 2, Veritor had PPA, NPA, and OPA values of 97.4, 98.1, and 98.1%, respectively, with Sofia 2. Discordant analysis showed one Lyra positive missed by Veritor and five Lyra positives missed by Sofia 2; one Veritor positive result was negative by Lyra. Veritor met FDA emergency use authorization (EUA) acceptance criteria for SARS-CoV-2 antigen testing for the 0 to 5 and 0 to 6 DSO ranges (PPA values of 83.9% and 82.4%, respectively). Veritor and Sofia 2 showed a high degree of agreement for SARS-CoV-2 detection. The Veritor test allows for more rapid COVID-19 testing utilizing easy-to-collect nasal swabs but demonstrated <100% PPA compared to PCR.

## Abstract (precis)

The clinical performance of the BD Veritor System for Rapid Detection of SARS-CoV-2 nucleocapsid antigen (Veritor), a chromatographic immunoassay used for SARS-CoV-2 point-of-care testing, was evaluated using nasal specimens from individuals with COVID-19 symptoms. Two studies were completed to determine clinical performance. In the first study, nasal specimens and either nasopharyngeal or oropharyngeal specimens from 251 participants with COVID-19 symptoms (≤7 days from symptom onset [DSO], ≥18 years of age) were utilized to compare Veritor with the Lyra SARS-CoV-2 PCR assay (Lyra).

## INTRODUCTION

In response to the COVID-19 pandemic, an emphasis has been placed on SARS-CoV-2 diagnostic testing for symptomatic individuals (1). Although laboratory-based PCR testing is considered the laboratory reference standard for COVID-19 diagnosis, it is associated with some drawbacks, including limitations in capacity (2, 3), which can lead to prolonged turnaround time (at best 24 h when sample shipment is considered). In addition, dedicated staff and automated platforms are usually required to provide an effective turnaround time and optimized patient management (4). Shortages of reagents and swabs for sample acquisition have also limited the capacity associated with molecular testing (5, 6).

In February 2020, the World Health Organization (WHO) identified point-of-care (POC) testing as a number one priority to address the COVID-19 pandemic (7). Importantly, recent work has demonstrated that delays in test reporting can negatively impact the value of isolation as a control measure to reduce the spread of SARS-CoV-2 (8). The relatively small investment in resources and expertise required to perform POC testing makes it ideal for use in decentralized health care settings (4).

This is the first detailed report that describes the results from a study supporting U.S. Food and Drug Administration (FDA) emergency use authorization (EUA) for a SARS-CoV-2 antigen test. Here, performance of the BD Veritor System for Rapid Detection of SARS-CoV-2 (Veritor test) was determined using nasal swab specimens from a population of COVID-19 symptomatic individuals. The Lyra SARS-CoV-2 assay (Lyra assay) was utilized as the laboratory reference standard. The results are also shown here, from an additional study, which directly compares the Veritor test to another SARS-CoV-2 antigen test, the Quidel Sofia 2 SARS Antigen FIA test (Sofia 2 test). Of importance, the population utilized for Veritor test comparison to the laboratory reference standard and the Sofia test reflects that which POC antigen testing is intended for use (i.e., outpatient settings, walk-in clinics, drive-through testing facilities, etc.).

## MATERIALS AND METHODS

### Study design.

Both studies described here involved a prospective collection of upper respiratory specimens. Eligible participants were ≥18 years of age and presented with one or more self-reported COVID-19 signs or symptoms (9, 10). Individuals were excluded if a nasal swab was collected as part of the standard of care (SOC). Demographic and health care-related information was collected (e.g., symptomology, health history, etc.). No study procedures were performed without an informed consent process or signature of a consent form. This research was performed in accordance with Good Clinical Practice guidelines and the Declaration of Helsinki. This article was prepared according to STARD guidelines for diagnostic accuracy studies reporting (11).

### Specimen collection.

**(i) Study 1 (EUA Veritor/Lyra comparison).** The first study was utilized to determine whether the Veritor test met FDA EUA criteria for detection of SARS-CoV-2 in COVID-19 symptomatic individuals (within ≤7 days from symptom onset [DSO]). Collection of specimens from 260 participants occurred across 21 geographically diverse study sites between 5 and 11 June 2020. Specimens for the Veritor test were from clinician-collected nasal specimens using regular-tipped flocked swabs (Becton, Dickinson and Company, BD Life Sciences—Integrated Diagnostics Solutions, Sparks, MD) inserted approximately 2.5 cm up the nostril (from the edge of the nostril). The swab was rolled five times along the mucosa of the nostril to ensure that sufficient mucus and cells were collected; the process was repeated in the other nostril using the same swab.

Lyra assay specimens came from nasopharyngeal (NP) or oropharyngeal (OP) swabs; SOC OP or NP swabs were taken before any study swabs. If an NP swab was collected as part of SOC, the participant had the option of having an OP study swab taken in lieu of a second NP swab. All NP (*n* = 217) or OP (*n* = 34) specimens were clinician collected. Swab collection for participants occurred in the following order: (i) SOC swab specimen, (ii) nasal swab specimen, and (iii) NP or OP swab specimen. Reference testing was performed at TriCore Reference Laboratories, while the Veritor testing was performed internally at Becton, Dickinson and Company (BD Life Sciences—Integrated Diagnostics Solutions, San Diego, CA).

**(ii) Study 2 (Veritor/Sofia 2 comparison).** The second study involved a comparison of Veritor test performance to the Sofia 2 test for SARS-CoV-2 detection, run with the Sofia 2 analyzer. Collection occurred from 377 participants with symptoms of COVID-19 (≤5 DSO) from five study sites in the United States. Specimen collection for Veritor testing was performed as described above. For Sofia 2 testing, clinician-collected nasal specimens were obtained using methods and swabs described in the instructions for use (IFU; Puritan regular foam swabs; Puritan, Guilford, ME). The specimens were obtained from a single nostril (with the most visible secretion) using gentle rotation. In some cases, due to an update in the Sofia 2 IFU, participants were instructed to blow their nose prior to nasal swab specimen collection (nose blowing is off-label for the Veritor test). NP swab specimen collection for the Lyra assay (only for Veritor/Sofia 2 discordant testing) was performed as described above. Swab collection for participants occurred in the following order: (i) SOC swab specimen, (ii) nasal swab specimen, and (iii) NP swab specimen. Testing for Veritor, Sofia 2, and discordant Lyra assay, was performed at TriCore Reference Laboratories. In order to minimize the impact of collection order on performance, swab collection for the Veritor and Sofia tests was randomized.

### Test procedures.

Swabs were shipped for testing on dry ice (–70°C); nasal swabs were shipped dry, and OP/NP swabs were shipped in universal viral transport medium. All testing was conducted with all personnel blinded to all other test results.

The Veritor and Sofia 2 tests are chromatographic, immunoassay-based platforms. The tests were performed according to the manufacturer’s IFU (BD Life Sciences—Integrated Diagnostic Solutions, San Diego, CA [12], and Quidel Corporation, Athens, OH [13], respectively), with the exception of transport of the swabs as frozen specimens for both assays. Internal validation showed no significant change in the performance of either test using frozen versus fresh specimens. Swabs were removed from −70°C storage ≤5 h prior to the time of testing. Swabs were placed at 2 to 8°C for ≥2 h and then at room temperature for 10 to 30 min prior to testing.

For specimen extraction prior to Veritor or Sofia 2 testing, the swabs were added to each respective extraction buffer tubes and mixed for at least 15 to 30 s or 1 min, respectively. The extraction buffer/specimen mixture from each test was then added to the sample well of the corresponding test cartridge to initiate the testing. After the assays proceeded for 15 min, the test cartridges were inserted into either the Veritor or Sofia 2 analyzer to obtain results.

The Lyra assay was performed according to the manufacturer’s IFU (Quidel Corporation, Athens, OH) (14). When using the NucliSENS easyMAG and the Applied Biosystem 7500 Fast Dx Real-Time PCR instrument, the Lyra assay reports cycle number in a manner that omits the first 10 cycles; here, the cycle numbers for the Lyra assay are reported with the first 10 cycles included. The BD MAX real-time SARS-CoV-2 PCR assay (MAX assay) was used for discordant testing on residual nasal swabs following Veritor and Lyra testing in study 1. The MAX assay was performed according to the manufacturer’s IFU (Becton, Dickinson and Company, BD Life Sciences—Integrated Diagnostic Solutions, Sparks, MD) (15).

### Data collection and statistical analyses.

The primary outcome measures for this study were positive, negative, and overall percent agreement (PPA, NPA, and OPA, respectively) point estimates for the Veritor test compared to results from the Lyra assay in study 1 and for the Veritor test compared to the Sofia 2 test in study 2.

For study 1, the acceptance criterion was a point estimate of ≥80% PPA of the Veritor test compared to the Lyra assay; clinical evaluation required contiguous enrollment to a minimum of 30 prospectively collected positive specimens as specified in the Antigen Template for Manufacturers (11 May 2020) for EUA submissions to the U.S. FDA (16). Based on an estimated 10% prevalence rate, it was necessary to enroll approximately 300 participants to achieve the required number of positives.

For study 1, the positive predictive value, negative predictive value, and accuracy were also calculated as secondary outcomes (17). In addition, a two-sample *t* test (two-tailed) was used to compare means between Lyra assay positive threshold cycle (*C_T_*) values on specimens matched to Veritor negative and positive test results for SARS-CoV-2 in study 1.

## RESULTS

### Study 1 (EUA study).

**(i) Participant reconciliation, demographics, and COVID-19 symptomology.** The mean and median age of the participants (45.0 and 43 years, respectively) were close (see Table S1 in the supplemental material). More than half (64.2%) of the participants were female. By race, the largest proportion of participants were white, followed by black, and then Asian. Approximately 40% were Hispanic or Latino. Cough was the most-reported symptom from participants, followed by muscle pain and then headache. While the drive-through/tent and outpatient clinic collection site categories represented approximately three-fourths of the collection sites, the research clinic category had the highest positivity rate (22.5%). The mean for DSO among the participants was 3.2 days (see Table S1). From 260 participants, six participants/participant specimen sets were removed due to inclusion/exclusion criteria noncompliance, and three were removed due to invalid specimens/results. Thus, 251 evaluable nasal specimens (each paired with either OP or NP specimens) were included (see Fig. S1a in the supplemental material).

**FIG 1:**
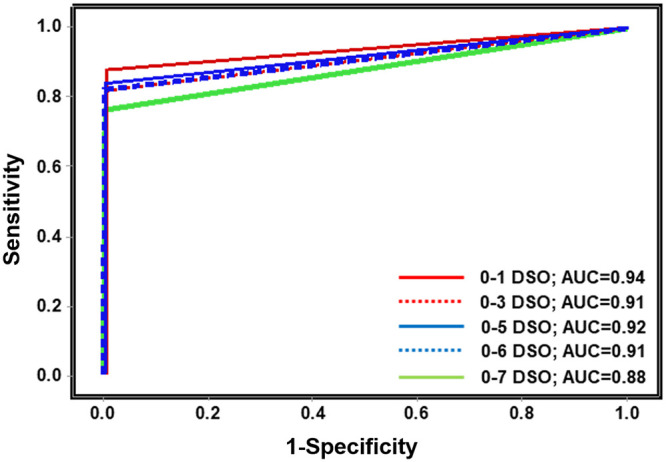
Veritor test performance results are plotted as a receiver-operator curve with sensitivity (corresponding to positive percent agreement) on the *y* axis and 1-specificity (corresponding to 1-negative percent agreement) on the *x* axis. Five lines, representing 0 to 1 DSO, 0 to 3 DSO, 0 to 5 DSO, 0 to 6 DSO, and 0 to 7 DSO are shown. Also shown are the area under the curve (AUC) values. Abbreviations: DSO, days from symptom onset; AUC, area under the curve.

**FIG 2:**
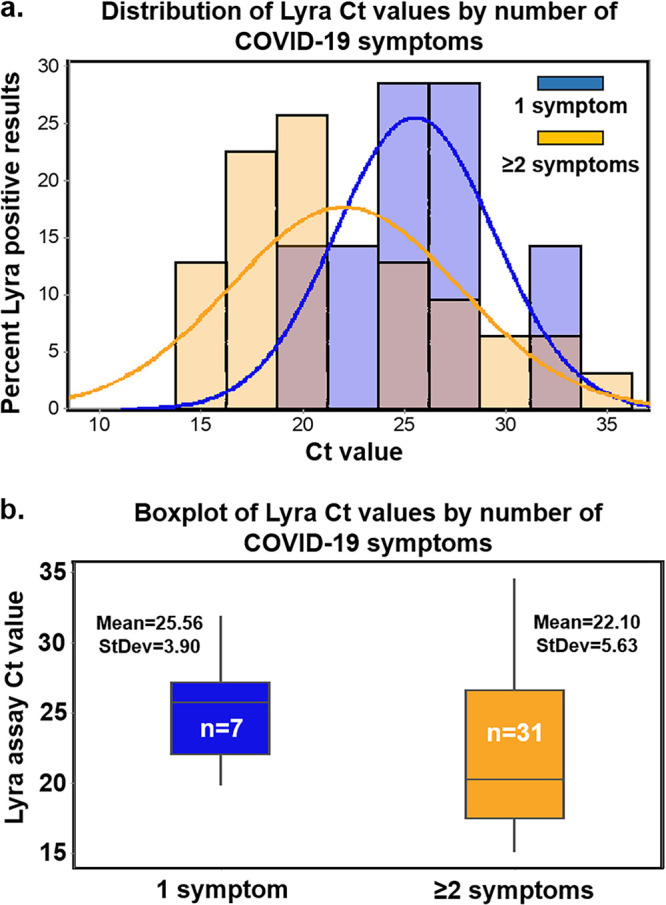
(a) The distribution of *C_T_* values corresponding to the 38 specimens that were positive by the Lyra assay (from specimens collected from participants, 0 to 7 DSO) following stratification by number of symptoms. *C_T_* score distribution for specimens matched to 1 symptom is shown in blue, while those matched to ≥2 symptoms are shown in orange; the pink color indicates blue/orange overlap. (b) The mean *C_T_* values (and standard deviation) are shown for the ≥2-symptom specimens (*n* = 31; mean = 22.10, standard deviation = 5.63) and the 1-symptom specimens (*n* = 7; mean = 25.56, standard deviation = 3.90). A two-sample *t* test (two-tailed) analysis indicated nonsignificant difference between the means (*P* = 0.077; mean difference of 3.46; [95% CI = −0.43 to 7.36]).

**(ii) Veritor test performance and discordant reconciliation.** Performance values for the Veritor test are indicated by DSO, for participants providing valid specimens (Table 1). The 0 to 5 DSO range was the shortest range tested to have a PPA value above 80% and include at least 30 reference positive results. The 0 to 6 DSO range also met PPA value acceptance criteria. The NPA for the Veritor test was 100% for the 0 to 1 to the 0 to 5 DSO ranges; however, the NPA value for the 0 to 6 and 0 to 7 DSO ranges was 99.5% (95% confidence interval [CI] = 97.4 to 99.9) (Table 1). The area under the curve (AUC) values associated with Veritor test performance for the 0 to 1 through the 0 to 6 DSO ranges were >0.9; the AUC value for the 0 to 7 DSO range was 0.88 (Table 1 and Fig. 1). Performance values for the Veritor test compared to the Lyra assay were analyzed by number of symptoms, as reported by participants during sample collection. As shown in Table 2, PPA point estimates were higher for the Veritor test when stratified by ≥2 symptoms versus 1 symptom for both the 0 to 5 DSO range (88.0 and 66.7%, respectively) and the 0 to 6 DSO range (88.9 and 57.1%, respectively). In addition, stratification of Lyra *C_T_* scores (for the 38 positive reference specimens represented in the entire 0 to 7 DSO range) by 1 versus ≥2 symptoms showed overlapping distributions that were offset, with the 1 symptom *C_T_* score distribution shifted toward higher *C_T_* values (Fig. 2a). The mean *C_T_* for the 1 symptom group (25.56), although not statistically different (*P* = 0.077) from the ≥2 symptom mean *C_T_* value (22.10), showed a trend toward having a higher value by approximately 3 cycles, an order of magnitude (Fig. 2b).

**TABLE 1:** Veritor test performance at 1 through 7 DSO^*a*^

Performance^*b*^	1 DSO	2 DSO	3 DSO	4 DSO	5 DSO^*c*^	6 DSO	7 DSO
% agreement (95% CI)							
PPA	87.5 (52.9–97.8)	85.0 (64.0–94.8)	81.8 (61.5–92.7)	85.2 (67.5–94.1)	83.9 (67.4–92.9)	82.4 (66.5–91.7)	76.3 (60.8–87.0)
NPA	100 (88.6–100)	100 (95.1–100)	100 (97.1–100)	100 (97.7–100)	100 (98.1–100)	99.5 (97.4–99.9)	99.5 (97.4–99.9)
OPA	97.4 (86.5–99.5)	96.8 (91.1–98.9)	97.3 (93.3–99.0)	97.9 (94.7–99.2)	97.8 (94.9–99.1)	97.1 (94.2–98.6)	96.0 (92.8–97.8)
AUC	0.94	0.93	0.91	0.93	0.92	0.91	0.88
							
True positives (*n*)							
Incident	7	10	1	5	3	2	1
Cumulative	7	17	18	23	26	28	29
							
False negatives (*n*)							
Incident	1	2	1	0	1	1	3
Cumulative	1	3	4	4	5	6	9
							
True negatives (*n*)							
Incident	30	45	52	35	33	15	2
Cumulative	30	75	127	162	195	210	212
							
False positives (*n*)							
Incident	0	0	0	0	0	1	0
Cumulative	0	0	0	0	0	1	1
							
Total (*n*)	38	95	149	189	226	245	251

a Abbreviations: DSO, days from symptom onset; PPA, positive percent agreement; NPA, negative percent agreement; OPA, overall percent agreement; AUC, area under the curve. CI, confidence interval; *n*, number of results.

b Performance of Veritor test compared to the Lyra assay as a reference.

c The Veritor test is FDA authorized for detection of SARS-CoV-2 only in individuals that are 0 to 5 DSO.

**TABLE 2:** Veritor test performance by number of symptoms at 0 to 5 and 0 to 6 DSO^*a*^

Performance^*b*^	0–5 DSO		0–6 DSO	
	1 symptom	≥2 symptoms	1 symptom	≥2 symptoms
% agreement (95% CI)				
PPA	66.7 (30.0–90.3)	88.0 (70.0–95.8)	57.1 (25.0–84.2)	88.9 (71.9–96.1)
NPA	100 (95.7–100)	100 (96.6–100)	100 (95.8–100)	99.2 (95.6–99.9)
OPA	97.8 (92.3–99.4)	97.8 (93.7–99.2)	96.8 (91.0–98.9)	97.4 (93.4–99.0)
				
No. of results				
True positives	4	22	4	24
False negatives	2	3	3	3
True negatives	85	110	87	123
False positives	0	0	0	1
Total	91	135	94	151

a Abbreviations: DSO, days from symptom onset; PPA, positive percent agreement; NPA, negative percent agreement; OPA, overall percent agreement; CI, confidence interval.

b Performance of Veritor test compared to the Lyra assay as a reference.

**FIG 3:**
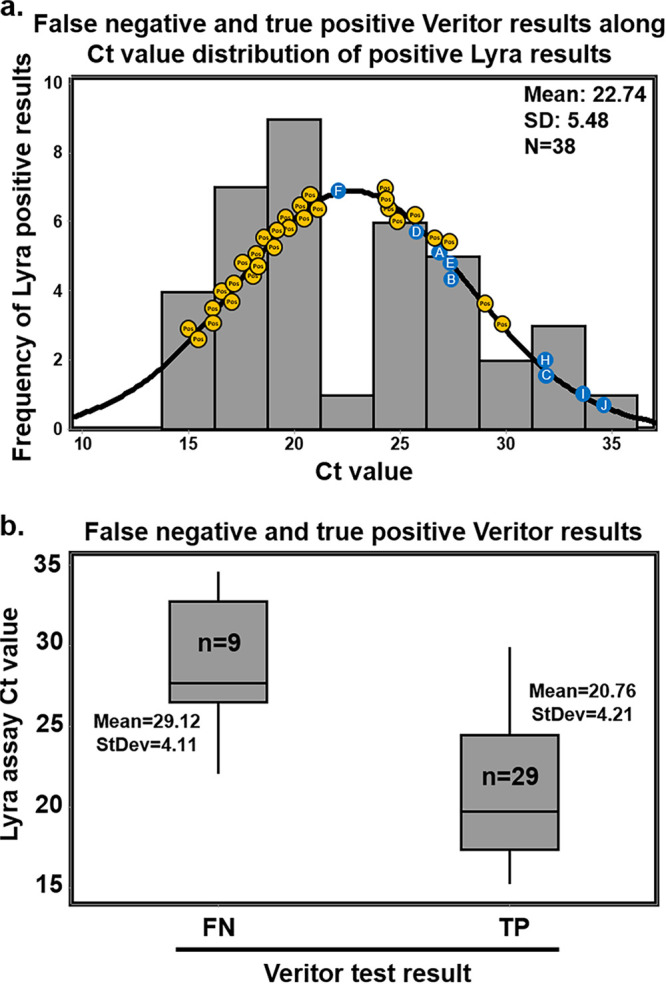
(a) The distribution of *C_T_* values corresponding to the 38 specimens that were positive by the Lyra assay (from specimens collected from participants, 0 to 7 DSO). Plotted along the fitted distribution line are the 29 true-positive Veritor results (orange circles) and the nine participant designations (letters superimposed onto blue circles), corresponding to those in Table 3, that represent the Veritor false-negative results matched to Lyra assay *C_T_* value. (b) The mean *C_T_* values (and standard deviation) are shown for the 29 true-positive (20.76 and 4.21, respectively) and the 9 false-negative (29.12 and 4.11, respectively) Veritor test results. A two-sample *t* test (two-tailed) analysis indicated a significantly higher mean Lyra assay *C_T_* value for specimens matched to the 9 Veritor test false-negative results compared to those matched to the 29 true positive results (*P* < 0.001; mean difference of 8.36; [95% CI = 4.95 to 11.77]).

Eight of the nine false-negative specimens by the Veritor test were from participants that had Lyra assay *C_T_* values which were greater than the mean Lyra *C_T_* value (22.74); the ninth fell just below the mean value (*C_T_* score of 22.04) (Fig. 3a). The Lyra assay mean *C_T_* value for the 29 specimens corresponding to true positive results for the Veritor test was 20.76 (standard deviation of 4.21). The Lyra assay mean *C_T_* value for the nine specimens corresponding to Veritor test discordant (negative) results was 29.12 (standard deviation of 4.11). This resulted in a statistically significant mean difference of 8.36 (*P* < 0.001; two-sample *t* test [two-tailed]; 95% CI = 4.95, 11.77) (Fig. 3b). Discordant analysis by testing on the MAX assay showed a positive result for only two of the nine Veritor test-negative samples (Table 3). From the remaining seven discordant findings, six were associated with a negative MAX assay result and one was associated with an unresolved result (no detection of internal control in the MAX assay).

**TABLE 3:** Discordant analysis for specimens associated with disagreement between the Veritor test and the Lyra assay^*a*^

DSO	Participant	False negative (*n*)		False positive (*n*)		Lyra result (*C_T_*)	Veritor result^*b*^	MAX result (*C_T_*)	Serology result^*c*^
		Incident	Cumulative	Incident	Cumulative				
0–1	A	1	1	0	0	POS (27.21)	NEG*	NEG	NA
0−2	B	1	2	0	0	POS (27.60)	NEG*	NEG	NA
	C	1	3	0	0	POS (31.90)	NEG*	NEG	NA
0–3	D	1	4	0	0	POS (25.72)	NEG	POS (34.02)	POS: IgM and IgG
0–4	NA	0	4	0	0	NA	NA	NA	NA
0–5	E	1	5	0	0	POS (27.56)	NEG*	NEG	NA
0−6	F	1	6	0	0	POS (22.04)	NEG	UNR^*d*^	NA
	G	0	6	1	1	NEG (NA)	POS	NEG	NA
0−7	H	1	7	0	1	POS (31.84)	NEG	POS (32.72)	POS: IgM and IgG
	I	1	8	0	1	POS (33.57)	NEG*	NEG	POS: IgM and IgG
	J	1	9	0	1	POS (34.60)	NEG*	NEG	NA

a Abbreviations: DSO, days from symptom onset; FN, false negative; FP, false positive; POS, positive; NEG, negative; NA, not available; *n*, number of findings.

b *, Agreement of Veritor test with the MAX assay for a negative result for SARS-CoV-2.

c Serology testing was done as part of the standard of care prior to study-related activities.

d UNR, unresolved. The RNase P result (internal control) in the MAX assay was negative, suggesting no presence of human material on the nasal swab.

Positive predictive values (PPV) for the Veritor test were 100% for the 0 to 1 DSO through the 0 to 5 DSO ranges. There was only a single Veritor test-positive/Lyra assay-negative discordant result in the study, which occurred in the 0 to 6 DSO group and resulted in PPV point estimates of 96.6 and 96.7% for the 0 to 6 and 0 to 7 DSO ranges, respectively. The negative predictive values (NPV) for the 0 to 1 to the 0 to 6 DSO groups ranged from 96.8 to 97.2. At 0 to 7 DSO, the NPV was 95.9 (see Fig. S2).

### Study 2 (Veritor/Sofia 2 test comparison study).

**(i) Participant reconciliation, demographics, and COVID-19 symptomology.** From 377 participants, four specimen sets were removed due to noncompliance with either inclusion or exclusion criteria, 16 were removed due to inappropriate sample collection/handling/transport, or invalid test results. There were 361 evaluable specimens included in analysis for this study (see Fig. S1b). The mean and median ages of the participants (45.4 and 44 years, respectively) were similar. Headache, cough, muscle pain, sore throat, and chills were the five most common symptoms reported (see Table S2).

**(ii) Veritor test performance and discordant reconciliation.** The PPA, NPA, and OPA for the Veritor test compared to the Sofia 2 test using specimens at the 0 to 5 DSO range were 97.4 (95% CI = 86.5 to 99.5), 98.1 (95% CI = 96.0 to 99.1), and 98.1 (95% CI = 96.1 to 99.1), respectively (Table 4). Of the seven discordant results, one was Veritor negative/Sofia 2 positive and was positive by the Lyra assay; six were Veritor positive/Sofia 2 negative, with 5 being positive by the Lyra assay and one being negative by the Lyra assay.

**TABLE 4:** Agreement between Veritor and Sofia 2 for detection of SARS-CoV-2^*a*^

Parameter	Result
% agreement (95% CI)	
PPA	97.4 (86.5–99.5)
NPA	98.1 (96.0–99.1)
OPA	98.1 (96.1–99.1)
	
Test finding (no.)	
Veritor (+)/Sofia 2 (+)	37
Veritor (–)/Sofia 2 (+)	1^*b*^
Veritor (+)/Sofia 2 (–)	6^*c*^
Veritor (–)/Sofia 2 (–)	317

a Abbreviations: PPA, positive percent agreement; NPA, negative percent agreement; OPA, overall percent agreement; CI, confidence interval.

b The one negative Veritor test/positive Sofia 2 test result was positive by Lyra assay discordant testing.

c Of the six positive Veritor test/negative Sofia 2 test results, five were positive and one was negative by Lyra assay discordant testing.

## DISCUSSION

Antigen-based immunoassay POC tests for SARS-CoV-2 can target multiple viral antigens, including spike or nucleocapsid protein in a cartridge-based, lateral flow format. Although it is too early to determine whether one target is advantageous over another, evidence supports the efficacy of nucleocapsid detection in these types of antigen-based assays (18, 19). Reports involving SARS and SARS-CoV-2 have demonstrated that the nucleocapsid protein is produced at high levels relative to the other viral proteins (20, 21). In addition, nucleocapsid detection was recently shown, albeit in a serology-based test, to result in higher sensitivity for detection of SARS-CoV-2 compared to spike protein detection (22).

Here, the Veritor test was required to achieve ≥80% PPA relative to the laboratory reference standard (with at least 30 positive specimens by reference) in order to be considered acceptable for FDA EUA. The Veritor test showed 83.9 and 82.4% PPA values for specimens from COVID-19 symptomatic participants that were 0 to 5 and 0 to 6 DSO, respectively. In addition, the AUC values for the 0 to 1 through the 0 to 6 DSO ranges were excellent (ranging from 0.91 to 0.94). The results presented here suggest that the Veritor test should be effective in settings that would benefit from POC testing (e.g., decentralized health care settings) in order to classify 0 to 5 or 0 to 6 DSO individuals as positive or negative for SARS-CoV-2 infection to support patient management.

There were 10 total discordant Lyra assay/Veritor test discordant results; 9 were Lyra assay positive but Veritor test negative, and 1 was Lyra assay negative but Veritor test positive. Discordant analysis for the 0 to 1 DSO through the 0 to 6 DSO specimens revealed one false-negative result (participant D from Table 3) that was associated with a high (34.02) *C_T_* value for the MAX assay (which, based on internal validation, has a limit of detection of 800 genomic RNA copies/ml; the same as the reported limit of detection for the Lyra assay).(14) Interestingly, participant D had a positive SOC serology result (both IgM and IgG), suggesting that the individual likely had a DSO greater than three. The nasal specimen from participant F had no detectable internal control (RNase P gene), suggesting a lack of integrity for this specimen. The remaining four participants (A, B, C, and E) had nasal specimens that were negative by the MAX assay, agreeing with the Veritor test. The false-positive (participant G) Veritor test result had a line value that was close to the positive cutoff and was therefore a low positive.

Here, the Veritor test had ≥96.0% PPV and NPV for detection of the SARS-CoV-2 nucleocapsid antigen at all DSO ranges tested. Plotted values demonstrate the dependence of Veritor test NPV on disease prevalence (see Table S3). Reflex testing (e.g., PCR-based testing) may be appropriate following a negative Veritor test result depending on the pretest probability and level of certainty required for patient management given medical history and future clinical action.

Discordant analysis for study 2 was performed using the Lyra assay and resulted in five Lyra and Veritor-positive/Sofia 2-negative, one Lyra- and Sofia 2-positive/Veritor-negative, and one Veritor-positive/Lyra- and Sofia 2-negative result. For the latter result, the apparent false positive was associated with a Veritor test value that was close to the positive cutoff; this low positive was the lowest positive Veritor value observed in study 2.

PCR-based assays for diagnostic applications are typically highly sensitive for detecting target analyte relative to other diagnostic methods. However, recent results challenge whether this is always advantageous in all diagnostic settings. Bullard et al. (23) and Wolfel et al. (24) recently showed PCR-positive results at time points corresponding with negative culture-based testing for active SARS-CoV-2. Importantly, this discrepancy between testing methods seems to emerge around 6 to 8 DSO (23, 24). In addition, Wolfel et al. show that the presence of sgRNA, a molecular marker for replicating SARS-CoV-2 virus, peaks around 4 to 5 DSO and then decreases drastically by 6 to 7 DSO (24). Finally, antigen-based test accuracy improves significantly when specimens associated with reference PCR values of 31 to 40 *C_T_* are removed from analysis and only specimens matched with reference values of ≤30 Ct are included (19). Eight of the nine false-negative Veritor test results here were matched with Lyra assay *C_T_* values that were above the mean *C_T_* value for the 38 Lyra assay-positive results (four were approximately 10 cycles above). This, combined with the significant difference in Lyra-matched *C_T_* values for the 29 Veritor test true-positive and 9 Veritor test false-negative specimens, suggests that Veritor-to-Lyra concordance is indirectly proportional to the Lyra assay *C_T_* score.

While PCR-based testing is sensitive for target detection, other testing modalities (such as antigen-based testing) may also be informative and may help clinicians determine the peak time period during which infections are transmissible. However, more data are needed to establish the efficacy of antigen-based tests, such as Veritor or Sofia 2, for identifying contagious individuals—especially in the asymptomatic population. The Veritor and Sofia 2 tests are currently only authorized for individuals suspected of having a SARS-CoV-2 infection at 0 to 5 DSO. In addition, the high level of agreement observed between the Veritor and Sofia 2 tests is consistent with reported, similar limits of detection for SARS-CoV-2 (12, 13).

The difference in EUA labeled sensitivity for Sofia 2 (96.7%) versus Veritor (84%) was not supported by this study, probably due to spectrum differences in study design and patient populations in this study versus the Sofia 2 EUA study. The patient population chosen for this study was intended to reflect the performance of the Veritor test in clinical settings where decentralized POC testing such as antigen testing would be most appropriate. The study data presented here included a large proportion of specimens collected from clinical settings, such as drive-through testing, tents, and outpatient clinics, and therefore likely includes individuals with milder severity illness, compared to study populations that have been used to generate sensitivity estimates for other EUA antigen tests where enrollment included emergency department patients and hospitalized patients. Several publications have demonstrated an association between severe disease and higher viral loads, which could inflate antigen test sensitivity performance estimates compared to performance estimates generated in patients with milder disease (25–30). The finding in this study of an observed *C_T_* score shift for subjects with 1 symptom versus ≥2 symptoms also supports the possibility that there may even be differences in viral load according to disease severity even among patients with milder disease. The analyses here (Table 2 and Fig. 2) suggest that ≥2 symptoms also demonstrated a higher PPA than 1 symptom alone, which is reflective of the trend toward lower *C_T_* scores (higher viral load) for specimens from participants with ≥2 symptoms.

### Limitations.

The data presented here are applicable to symptomatic patients and performance in asymptomatic patients cannot be determined based on the results from this study. Nasal swabs were collected after the SOC clinical swab, which may have compromised the integrity of the nasal study swab (e.g., it may have introduced infected cells from the nasopharynx into the anterior nares). For the Lyra assay, results came from more than one swab specimen type (either OP or NP). This could have affected the reproducibility for Lyra assay results. However, there were only 34 OP swabs collected during the EUA study, and only one OP was positive by the Lyra assay. Since these numbers are low, we do not believe that any differences that may exist between performance from the two swabs had a meaningful impact on the study results. Although the Veritor test was performed on nasal swab specimens, the Lyra assay was performed on either NP (or OP) swab specimens per FDA EUA requirements. Other EUA submissions (the LumiraDx SARS-CoV-2 Ag Test [Luminar test] and the Abbott BinaxNOW COVID-19 Ag CARD [Abbott test]) utilized nasal swab specimens for both the antigen test and the reference PCR assay. Furthermore, MAX assay results from the remnant Veritor nasal swab in this report agreed with negative Veritor results in 7 of 9 discordant specimens. Improved PPA for Veritor versus Lyra may have been achieved using paired nasal swab specimens in the EUA study.

The Sofia 2 assay in study 2 was performed on nasal swabs that were collected either with (*n* = 56; see Table S4) or without (*n* = 305; see Table S5) a nose blowing step prior to collection. The nose-blowing step was an addition to the Sofia 2 test IFU intended only to reduce the frequency of invalid results (by reducing the amount of mucosal or blood-derived inhibitors in the specimen) and was not included in order to alter the performance of the Sofia 2 test. Although the number is low for specimens with a pre-nose blowing step in study 2, the results here suggest that the nose-blowing step did not alter the overall performance of the Sofia 2 test in relation to the Veritor test.

### Conclusions.

The Veritor test met acceptance criteria for EUA for antigen testing (≥80% PPA point estimate) for the 0 to 5 and 0 to 6 DSO ranges in a population of 251 subjects. The 0 to 1 through the 0 to 6 DSO ranges had AUC values of ≥0.90, suggesting that it is a reliable point-of-care test. The results here suggest that number of symptoms may influence the sensitivity of antigen-based POC testing. In additional testing, Veritor returned 43 positive results and Sofia 2 returned 37 positive results from a population of 361 subjects. The speed (15-min run time) and performance of antigen tests for SARS-CoV-2 detection should facilitate rapid and reliable results for COVID-19 diagnosis. Importantly, this POC test is run on nasal swab specimens, which are relatively easy and safe to collect. This study generated point estimates from a population that represents the most appropriate intended use population and thus can be used to inform proper patient management. In addition, the Veritor test should have a significant impact in decentralized health care settings where requirements for larger-scale PCR-based tests are harder to meet or result in extended turnaround times.

## Supplementary Material

Supplemental file 1
