# Supplementary material for: Clinical Evaluation of BD Veritor SARS-CoV-2 Point-of-Care Test Performance Compared to PCR-Based Testing and versus the Sofia 2 SARS Antigen Point-of-Care Test
Source: J Clin Microbiol. 2020 Dec 17;59(1):e02338-20. doi: 10.1128/JCM.02338-20 (PMC7771450; doi:10.1128/JCM.02338-20)
Supplement: Supplemental file 1 [file JCM.02338-20-s0001.pdf]

Supplemental Material
TABLE S1

Table S1. Study 1 (EUA) participant demographics (N=254)

**Age, years**	**Value**
Mean	45.0
SD	16.6
Median	43.0
Min	18
Max	90
**Gender, % (n)**	
Female	64.2 (163)
Male	35.8 (91)
**Race, % (n)**	
Asian	5.9 (15)
Black	10.2 (26)
White	56.7 (144)
Other	27.2 (69)
**Ethnicity, % (n)**	
Hispanic or Latino	40.6 (103)
Not Hispanic or Latino	55.9 (142)
Not reported	3.5 (9)
**Symptoms, % (n)**	
Cough	43.3 (110)
Muscle pain	38.6 (98)
Headache	37.4 (95)
Sore throat	35.4 (90)
Fever	30.7 (78)
Shortness of breath	20.5 (52)
Chills	17.7 (45)
New loss of taste or smell	4.7 (12)
Repeated shaking with chills	4.3 (11)
Other[a]	24.0 (61)
**Collection site**	
**All Sites**[b, c]	21
**Drive through/tent** (% of total sites)	23.8 (5/21)
Total specimens collected	42
% REF positive of total collected	2.4 (1/47)
**Outpatient clinic** (% of total sites)	52.4 (11/21)
Total specimens collected	74
% REF positive of total collected	24.3 (18/74)
**Research clinic** (% of total sites)	19.0 (4/21)
Total specimens collected	72
% REF positive of total collected	22.5 (16/71)
**Skilled nursing facility** (% of total sites)	4.8 (1/21)
Total specimens collected	66
% REF positive of total collected	4.5 (3/66)
**Overall positivity rate**	15.1 (38/252)
**Mean DSO**	3.2
**Median DSO**	3.0
**Mode DSO**	2.0

Abbreviations: SD, standard deviation; MIN, minimum; Max, maximum; DSO, days from symptom onset; REF, reference test

[a]"Other" symptoms include gastrointestinal issues, fatigue, chest pain, rhinorrhea, and nasal congestion

[b]Collection sites reside in the following states: Arizona, California, Florida (7), Georgia, Iowa, Louisiana, North Carolina, Nevada (2), Ohio (3), South Carolina, Texas, Utah

[c]% REF positive of total collected values were calculated from specmins only with valid Lyra assay results (n=252)

TABLE S2

Table S2. Study 2 (Veritor vs Sofia 2 comparison) participant demographics (N=373)

**Age, years**	**Value**
Mean	45.4
SD	16.4
Median	44
Min	18
Max	98
**Gender, % (n)**	
Female	59.5 (222)
Male	40.5 (151)
**Race, % (n)**	
Asian	0.3 (1)
Black	16.1 (60)
White	69.4 (259)
Other	14.2 (53)
**Ethnicity, % (n)**	
Hispanic or Latino	66.2 (247)
Not Hispanic or Latino	33.8 (126)
**Symptoms, % (n)**	
Cough	56.3 (210)
Muscle pain	53.9 (201)
Headache	67.8 (253)
Sore throat	52.8 (197)
Fever	34.3 (128)
Shortness of breath	13.7 (51)
Chills	37.5 (140)
New loss of taste or smell	17.7 (66)
Repeated shaking with chills	1.6 (6)
Other[a]	43.7 (163)
**Collection site**	
**All Sites**	5
**Drive through/tent** (% of total sites)	20.0 (1/5)
Veritor positivity rate (%)	11.1 (1/9)
Sofia 2 positivity rate (%)	11.1 (1/9)
**Outpatient clinic** (% of total sites)	20.0 (1/5)
Veritor positivity rate (%)	8.2 (4/49)
Sofia 2 positivity rate (%)	6.1 (3/49)
**Research clinic** (% of total sites)	40.0 (2/5)
Veritor positivity rate (%)	12.8 (38/298)
Sofia 2 positivity rate (%)	11.4 (34/297)[b]
**Urgent care** (% of total sites)	20.0 (1/5)
Veritor positivity rate (%)	0.0 (0/7)
Sofia 2 positivity rate (%)	0.0 (0/7)
**Overall (either test) positivity rate**	12.1 (44/364)
**Mean DSO**	3.0
**Median DSO**	3.0
**Mode DSO**	4.0

Abbreviations: SD, standard deviation; MIN, minimum; Max, maximum; DSO, days from symptom onset; REF, reference test

[a]"Other" symptoms include shaking, gastrointestinal issues, fatigue, chest pain, rhinorrhea, and nasal congestion

[b]Of the 298 specimens, one was Sofia 2 invalid

TABLE S3

Table S3. Positive and negative predictive values for the Veritor test compared to the Lyra Assay

	Prevalence (%)[a]							
**0-5 DSO**	**1.0**	**2.0**	**5.0**	**10.0**	**15.0**	**20.0**	**25.0**	**30.0**
**PPV** (%, [95% CI])	100 [33.2, 100]	100 [50.1, 100]	100 [72.1, 100]	100 [84.5, 100]	100 [89.7, 100]	100 [92.5, 100]	100 [94.2, 100]	100 [96.2, 100]
**NPV** (%, [95% CI])	98.8 [99.6, 99.9]	99.7 [99.3, 99.9]	99.2 [98.3, 99.7]	98.2 [96.4, 99.4]	97.2 [94.4, 99.0]	96.1 [92.2, 98.7]	94.9 [89.9, 98.2]	93.5 [86.6, 97.0]

Abbreviations: DSO, days from symptom onset; PPV, positive predictive value; CI, confidence interval; NPV, negative predictive value

[a]Prevalence of SARS-CoV-2 among the 0-5 DSO participants

TABLE S4

Table S4. Agreement between Veritor and Sofia 2 (only collection involving nose blowing prior to nasal swab collection) for detection of SARS-CoV-2

**PPA** %, [95% CI]	100 (56.6, 100)
**NPA** %, [95% CI]	98.0 (89.7, 99.7)
**OPA** %, [95% CI]	98.2 (90.6, 99.7)
Veritor (+)/Sofia 2(+)	5
Veritor (-)/Sofia 2 (+)	0
Veritor (+)/Sofia 2 (-)	1[a]
Veritor (-)/Sofia 2 (-)	50

Abbreviations: PPA, positive percent agreement; NPA, negative percent agreement; OPA, overall percent agreement

[a]The 1 positive Veritor test/negative Sofia 2 test result was positive by Lyra assay discordant testing

TABLE S5

Table S5. Agreement between Veritor and Sofia 2 (nose blowing not included prior to nasal swab collection) for detection of SARS-CoV-2

**PPA** %, [95% CI]	97.0 (84.7, 99.5)
**NPA** %, [95% CI]	98.2 (95.8, 99.2)
**OPA** %, [95% CI]	98.0 (95.8, 99.1)
Veritor (+)/Sofia 2(+)	32
Veritor (-)/Sofia 2 (+)	1[a]
Veritor (+)/Sofia 2 (-)	5[b]
Veritor (-)/Sofia 2 (-)	267

Abbreviations: PPA, positive percent agreement; NPA, negative percent agreement; OPA, overall percent agreement

[a]The 1 negative Veritor test/positive Sofia 2 test result was positive by Lyra assay discordant testing

[b]Of the 5 positive Veritor test/negative Sofia 2 test results 4 were positive and 1 was negative by Lyra assay discordant testing

FIGURE S1

Figure S1a.

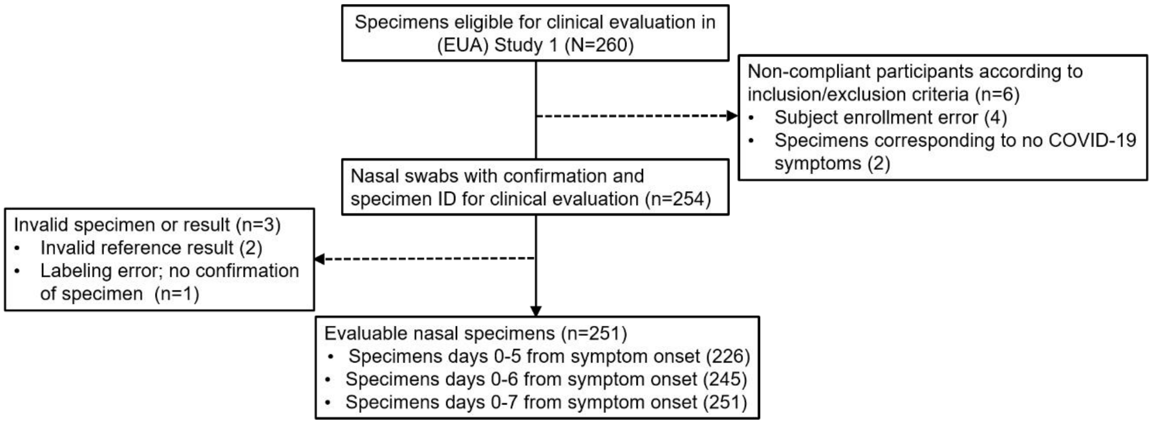

Figure S1b.

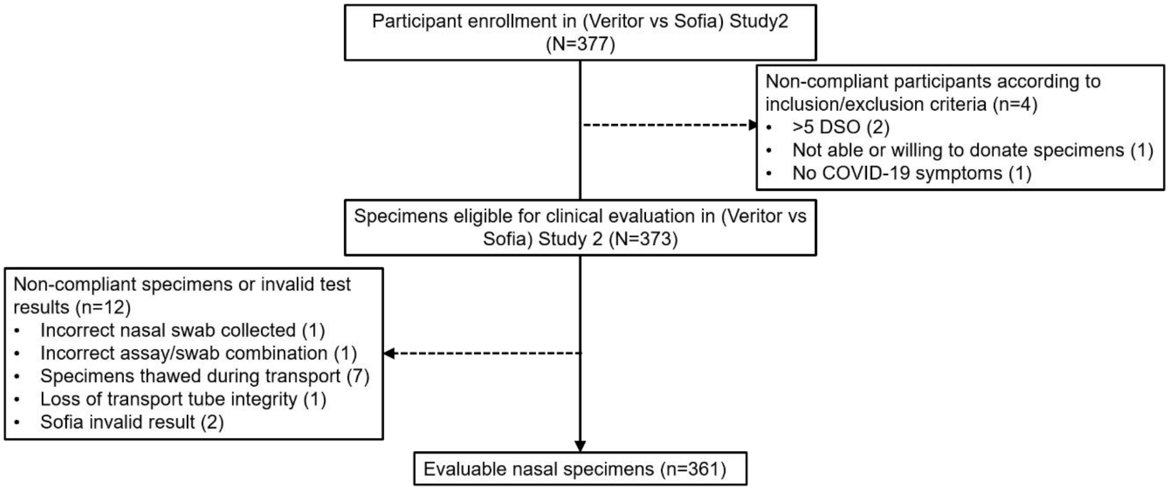

Figure S1. (a) Reconciliation during enrollment of swab specimens from participants, ≥18 years of age, with signs or symptoms of COVID-19 for study 1. **Abbreviations:** ID, identification; DSO, days from symptom onset **(b)** Reconciliation during enrollment of swab specimens from participants, ≥18 years of age, with signs or symptoms of COVID-19 for study 2. **Abbreviations:** DSO, days from symptom onset

FIGURE S2

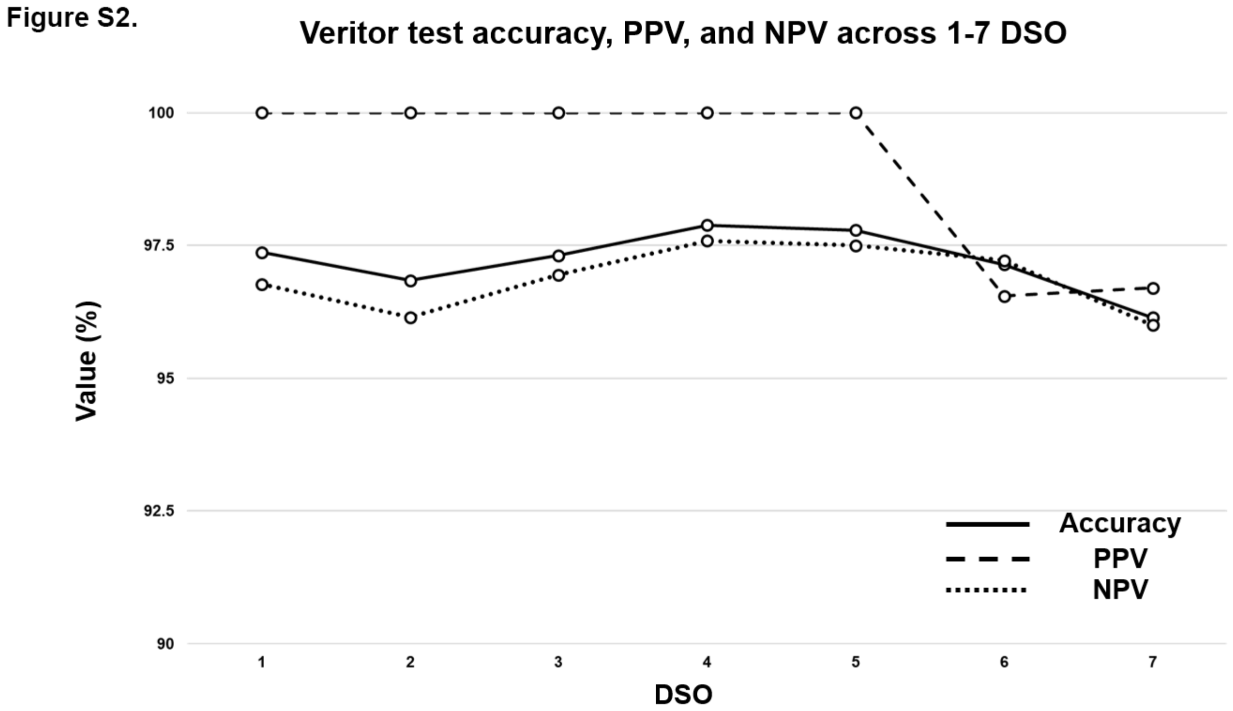

Figure S2. PPV, NPV, and test accuracy as a function of DSO for SARS-CoV-2 detection by the Veritor test. The point estimates for the three test values are plotted along the y-axis as percentages and the seven DSO ranges (0-1 to 0-7) reside along the x-axis. The SARS-CoV-2 prevalence value at each, respective, DSO range (based on positive reference results) was utilized for calculations and are as follows: 0-1 (21.1%), 0-2 (21.1%), 0-3 (14.8%), 0-4 (14.3%), 0-5 (13.7%), 0-6 (13.9%), and 0-7 (15.1%). Abbreviations: PPV, positive predictive value; NPV, negative predictive value; DSO, days from symptom onset.
